# Continuous Glucose Monitoring in the Real World Using Photosurveillance of #Dexcom on Instagram: Exploratory Mixed Methods Study

**DOI:** 10.2196/11024

**Published:** 2019-05-24

**Authors:** Michelle L Litchman, Sarah E Wawrzynski, Whitney S Woodruff, Joseph B Arrington, Quynh C Nguyen, Perry M Gee

**Affiliations:** 1 College of Nursing University of Utah Salt Lake City, UT United States; 2 Intermountain Healthcare Salt Lake City, UT United States; 3 Department of Biology University of Utah Salt Lake City, UT United States; 4 Department of Epidemiology and Biostatistics School of Public Health University of Maryland College Park, MD United States

**Keywords:** diabetes, continuous glucose monitoring, off-label use, social media, Instagram, photosurveillance

## Abstract

**Background:**

Individuals with diabetes are using social media as a method to share and gather information about their health via the diabetes online community. Infoveillance is one methodological approach to examine health care trends. However, infoveillance, while very effective in identifying many real-world health trends, may miss opportunities that use photographs as primary sources for data. We propose a new methodology, photosurveillance, in which photographs are analyzed to examine real-world trends.

**Objective:**

The purpose of this research is to (1) assess the use of photosurveillance as a research method to examine real-world trends in diabetes and (2) report on real-world use of continuous glucose monitoring (CGM) on Instagram.

**Methods:**

This exploratory mixed methods study examined all photographs posted on Instagram that were identified with the hashtag #dexcom over a 3-month period—December 2016 to February 2017. Photographs were coded by CGM location on the body. Original posts and corresponding comments were textually coded for length of CGM device wear and CGM failure and were analyzed for emerging themes.

**Results:**

A total of 2923 photographs were manually screened; 12.08% (353/2923) depicted a photograph with a CGM site location. The majority (225/353, 63.7%) of the photographs showed a CGM site in an off-label location, while 26.2% (92/353) were in an FDA-approved location (ie, abdomen) and 10.2% (36/353) were in an unidentifiable location. There were no significant differences in the number of likes or comments based on US Food and Drug Administration (FDA) approval. Five themes emerged from the analysis of original posts (N=353) and corresponding comments (N=2364): (1) endorsement of CGM as providing a sense of well-being; (2) reciprocating information, encouragement, and support; (3) reciprocating CGM-related frustrations; (4) life hacks to optimize CGM use; and (5) sharing and learning about off-label CGM activity.

**Conclusions:**

Our results indicate that individuals successfully used CGM in off-label locations, posting photos of these areas with greater frequency than of the abdomen, with no indication of sensor failure. While these photographs only capture a snapshot in time, these posts can be used to inform providers and industry leaders of real-world trends in CGM use. Additionally, there were instances in which sensors were worn beyond the FDA-approved 7-day period; however, they represented the minority in this study.

## Introduction

Individuals with diabetes are using social media as a method to share and gather information about their health via the diabetes online community. The diabetes online community is a grassroots collection of stakeholders affected by diabetes, including people with diabetes, caregivers (ie, parents of children with diabetes), health care providers, researchers, and industry who use Internet resources (ie, forums, Facebook, and Twitter) to discuss health-related issues [1-3]. Through shared experiences with virtual peers [2-7], diabetes online community users experience a sense of normalcy. As such, individuals are sharing information about a variety of aspects related to diabetes, including diabetes treatment options such as diabetes technology [2]. One social media site used within the diabetes online community that supports photo sharing is Instagram. If a picture is worth a thousand words, a diabetes-related photo posted on Instagram is worth a million *likes*. Diabetes online community users can optimize diabetes-related conversations on Instagram through the use of hashtags.

One technology that individuals with diabetes use to support self-care is real-time continuous glucose monitoring (CGM). CGM is comprised of a small sensor placed into the subcutaneous tissue, a transmitter, and a receiver or mobile phone that displays glucose levels and trends in real time. One of the two companies that supply CGM monitors for patient use is Dexcom. Currently, the Dexcom CGM system is approved by the US Food and Drug Administration (FDA) to be worn on the abdomen only for adults, and abdomen or buttocks for children. Further, the Dexcom G4 and G5 CGM systems are FDA approved to be worn for 7 days. Through clinical observation and anecdotal reports, patients are wearing CGM devices in off-label sites and extending the wear beyond 7 days. However, we do not know the extent to which CGM users are using off-label site locations or for how long.

Infoveillance is one methodological approach to examine health care trends [8,9]. This approach, which overlays Twitter data and geographic data, has been successful in identifying infectious diseases, such as influenza [10,11] and Zika [12], and suicide in veterans [13,14]. Infoveillance, while very effective in identifying many real-world health trends, may miss opportunities that use photographs as primary sources for data. We propose a new methodology, photosurveillance, in which photographs are analyzed to examine real-world trends. Photographs represent microreports of events of day-to-day life [15], such as diabetes management. Since some CGM users are sharing photographs of their diabetes experiences online, analyzing Instagram using a photosurveillance approach can help observe real-world use of CGM, including off-label activity. Off-label CGM activity may be difficult to track using other data collection methods, such as surveys or interviews, due to response bias. Thus, photosurveillance provides an opportunity to identify how individuals are using CGM in the real world without fear of disapproval from health care providers and researchers due to nonadherence to FDA guidelines. The purpose of this exploratory research is to (1) assess the use of photosurveillance as a research method to examine real-world trends in diabetes, and (2) report on real-world use of continuous glucose monitoring on Instagram. We anticipate that we will be able to successfully gather information about CGM use using photosurveillance. We hypothesize that there will be more off-label site locations compared to FDA-approved locations and that CGM wear greater than 7 days will be observed.

## Methods

### Dataset Acquisition and Sampling

This study was acknowledged as exempt by the University of Utah, Salt Lake City, Utah, ethics board. This descriptive study was conducted by hand-searching all photographs (N=2923) posted on Instagram identified with the hashtag #dexcom. A 3-month period was chosen, from December 2016 to February 2017, in order to keep the time-intensive process of hand-searching and coding data manageable, while allowing for an adequate sample size. During the search period, Dexcom was approved for 7-day wear. The hashtag #dexcom was chosen because it was more commonly used (N=46,105) when compared to other CGM-related hashtags—#dexcomg5, N=8350; #dexcomg4, N=6135; #dexcomcgm, N=1105; #enlitesensor, N=956; and #medtronicCGM, N=195. Instagram users include hashtags on their posts for searchability purposes. Additionally, #dexcom was frequently accompanied with other Dexcom-related hashtags, such as those just mentioned, resulting in duplication of posts. Thus, only one hashtag, #dexcom, was used in this analysis. Photographs were included for analysis if they depicted a CGM site and the original post was written in English (N=353). Photographs were excluded if they included a CGM site but were initiated from a company advertising their product (ie, adhesive decal).

### Photograph Analysis

Photosurveillance was conducted by categorizing photographs by site location, including FDA-approved sites (ie, in adults, this includes the abdomen only), off-label sites (ie, in adults, this includes the posterior arm, anterior arm, forearm, back, buttocks, thigh, and calf), and *other* sites. The *other* category represented photos of a CGM device on skin without clear body landmarks (ie, hand, foot, or belly button) that identified the exact location. Based on photograph content, we were not able to code for demographic factors, including age, gender, or diabetes type. Therefore, photographs of children and adults were not dichotomized, despite having different FDA approval for site locations. There were no duplicate photos. Two independent researchers (JA and WSW) input data into REDCap, a Web-based data capture program, to organize data [16]. Text from the original posts were analyzed to support data input. For example, in some instances, the photo would state that the CGM device was in a specific location, even if it was minutely visible. There were not instances in which photographs were recoded based on text content. A content analysis approach was utilized in which counts were categorized. Frequencies were used to describe the differences between FDA-approved and off-label activity.

### Post and Comment Analysis

Original posts (N=353) and corresponding comments (N=2364) were analyzed to examine discussions related to CGM activity. Comments were initially read and reread by two investigators (ML and JA) to develop the initial coding schema using an open-code approach. Successful CGM use was determined if the comment provided affirmation that a specific CGM site location worked for them (ie, CGM was reported to be most accurate, comfortable, or last the full length of the sensor, at minimum). CGM failure was categorized by inaccurate readings (ie, CGM readings did not match glucometer readings in a way that was significant to the user), CGM device ripping or falling off, too painful to continue wearing, bleeding impacting use, and unknown reasons. If the post or comment did not affirm success or failure, it was coded as *not applicable*. Post or comment mentions of off-label use of CGM wear more than 7 days were examined. Data were categorized for every 7 days worn beyond FDA approval (ie, 8-14 days, 15-21 days, etc). The frequency of *likes* (ie, “hearts”) and comments were analyzed using *t* tests.

### Qualitative Thematic Analysis

Subjective bias by the research team is an inherent possibility in qualitative research. Our team used *bracketing*, or the identification of one’s own bias and preunderstanding of CGM use for people with diabetes. We noted our individual prejudice and discussed this as a team prior to beginning this qualitative content analysis; we then re-evaluated and communicated frequently as a team to be sure our individual bias did not become part of the research findings [17,18]. Additional senior researchers who were experienced with qualitative content analysis independently verified the findings; this process was included in this study design [18]. Throughout the analysis, the open and axial coding processes were verified frequently with experienced researchers. 

Qualitative thematic analysis was conducted by examining each original post (N=353) and their corresponding comments (N=2364). Codes were used to organize similar data in order to identify discussions about FDA-approved and off-label CGM activity [19]. The codes were then systematically applied to all of the data using an open-code approach to capture any data that was not specific to FDA-approved or off-label CGM activity [19,20]. A matrix was created to maintain an audit trail [21]. Themes were developed from the data [22]. Content of data, and not code frequency, was used to assess data saturation [23]. To avoid risk of identification, no direct quotes were used in this manuscript.

## Results

### Photograph Analysis

Of the 2923 photographs examined during the study period, 12.08% (353/2923) depicted a photograph with a CGM site location. The remaining photos in the original sample (2570/2923, 87.92%) not further analyzed in this study included pictures of CGM trend data on receivers and/or mobile devices; glucometers; CGM supplies (eg, transmitters and sensors); references to food, beverages, or exercise; photos of a person or people; and memes. There were 194 unique users who posted the 353 unique photographs, each user posting 1-4 photographs each. Multiple posts by individuals were linked by an Instagram handle in the dataset. The majority (225/353, 63.7%) of the photographs showed CGM sites in off-label locations, while 26.2% (92/353) were in an FDA-approved location (ie, abdomen) and 10.2% (36/353) were in unidentifiable locations (ie, *unknown* category; see Table 1).

**Table 1:** Instagram photographs by CGM^a^ site (N=353).

CGM site location	Frequency, n (%)
Posterior arm	139 (39.4)
Abdomen (FDA^b^ approved)	92 (26.1)
Thigh	45 (12.7)
Unknown	36 (10.2)
Forearm	12 (3.4)
Back	10 (2.8)
Anterior arm	10 (2.8)
Calf	7 (2.0)
Buttocks	3 (0.8)

^a^CGM: continuous glucose monitoring.

^b^FDA: US Food and Drug Administration.

### Post and Comment Analysis

**Figure 1:**
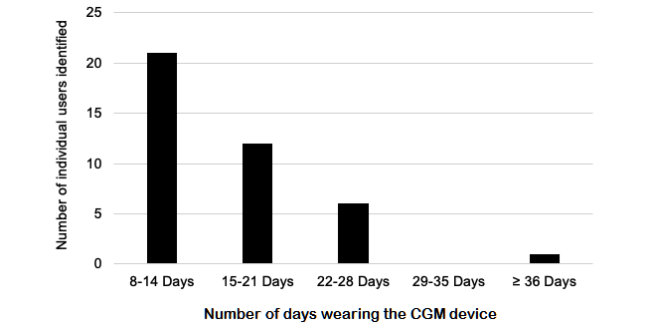
Reported continuous glucose monitoring (CGM) device use for more than 7 days (n=40).

Original posts (N=353) captured discussions about successful CGM use and failures. Additionally, some of the comments (N=2364) discussed CGM successes and failures, though not all; Table 2 reports sample sizes that include both the original post and comments for each site location. Success rates were similar in the abdomen and posterior arm (see Table 2). Inaccuracy concerns were noted more often in the abdomen, calf, and buttocks, although the sample size of the buttocks was relatively low (n=10). There were 40 individual users who noted wearing their CGM device successfully for more than 7 days (see Figure 1).

### Viral Measures

To examine the viral spread of CGM photos and associated comments indicating off-label or FDA-approved site locations, likes and comments were analyzed. There were no significant differences in the number of likes (FDA approved, N=9152; off-label, N=24,534; *P*=.85) or comments (FDA approved, N=707; off-label, N=1500; *P*=.16) based on FDA approval (see Table 3). However, the number of likes and comments were 3-4 times greater for off-label locations.

**Table 2:** CGM^a^ site discussions based on success and failure rates.

CGM site location	Success, n (%)	Inaccurate, n (%)	Ripped or fell off, n (%)	Too painful, n (%)	Bleeding, n (%)	Unknown reason, n (%)	Unable to determine, n (%)
Abdomen (N=178)	137 (77.4)	11 (6.2)	3 (1.7)	6 (3.4)	0 (0)	1 (0.6)	20 (11.2)
Posterior arm (N=273)	218 (79.9)	6 (2.2)	3 (1.1)	0 (0)	0 (0)	2 (0.7)	44 (16.1)
Thigh (N=198)	137 (69.2)	6 (3.0)	2 (1.0)	0 (0)	5 (2.5)	3 (1.5)	45 (22.7)
Back (N=42)	24 (57)	2 (5)	0 (0)	0 (0)	0 (0)	0 (0)	16 (38)
Calf (N=48)	20 (42)	3 (6)	0 (0)	0 (0)	1 (2)	0 (0)	24 (50)
Anterior arm (N=30)	19 (63)	0 (0)	0 (0)	0 (0)	1 (3)	0 (0)	10 (33)
Forearm (N=38)	22 (58)	0 (0)	1 (3)	0 (0)	0 (0)	0 (0)	15 (39)
Buttocks (N=10)	6 (60)	1 (10)	1 (10)	0 (0)	0 (0)	0 (0)	2 (20)

^a^CGM: continuous glucose monitoring.

**Table 3:** Engagement with off-label and FDA^a^-approved CGM^b^ site posts.

Engagement	Off-label, n	FDA approved, n	*P* value^c^
Likes	24,534	9152	.85
Comments	1500	707	.16

^a^FDA: US Food and Drug Administration.

^b^CGM: continuous glucose monitoring.

^c^*P* values are based on *t* tests.

### Qualitative Thematic Analysis

Five themes emerged from the analysis of original Instagram posts and the corresponding comments: (1) endorsement of CGM as providing a sense of well-being; (2) reciprocating information, encouragement, and support; (3) reciprocating CGM-related frustrations; (4) life hacks to optimize CGM use; and (5) sharing and learning about off-label CGM activity.

### Endorsement of Continuous Glucose Monitoring Providing a Sense of Well-Being

CGM was described as a “life saver.” Hypoglycemia alarms were valued more than hyperglycemia alarms with regard to immediate safety concerns. Several posts mentioned the CGM *share* feature providing peace of mind for loved ones. Care partners who visualized glucose levels provided support to those with diabetes who reported that they could confidently engage in day-to-day activities, such as exercise and sleep. In fact, individuals felt so strongly about CGM, they used social media as a platform to encourage others with diabetes to use the technology. Social media was also used to teach Instagram followers, who may not have had diabetes, about CGM technology.

Many users shared that using CGM allowed them to think less about their diabetes and confidently manage their health in a less intrusive way. Women who were already pregnant or trying to become pregnant described the value of CGM in having a healthy baby. One person posted, “We've been trying to get pregnant so it feels like such a victory to be closer to my goal! I almost cried [when my hemoglobin A1c was] at 6%!” In addition to CGM, women who were pregnant also described the value of diabetes educators in helping them make sense of the CGM data. Parents described the value of being able to observe glucose levels without having to be intrusive. One parent posted, “[My child’s CGM] means that I don't have to wake her up at 1am on nights that she eats pasta or lo mein to check her levels. What a gift.” Both adults with diabetes and parents of children with diabetes valued CGM as improving their quality of life.

Individuals also mentioned that CGM use reduced their worry by alerting them early to trends of high or low glucose levels. Although, there were also mentions about alarms being too frequent and some described alarm fatigue. CGM, in some cases, also provided insight into daily trends like overnight hypo- or hyperglycemia and caused them to change their insulin doses or behaviors in an attempt to remedy out-of-range glucose levels. Several posts attributed the use of CGM to a reduction in users’ glycosylated hemoglobin A1c levels and even in prolonging their life.

### Reciprocating Information, Encouragement, and Support

Posters and commenters used Instagram as a space to share information based on experiential knowledge. Successes, frustrations, and daily life—#t1dlookslikeme and #thisiswhatdiabeteslookslike—with diabetes were discussed, all of which resulted in encouragement, support, and sometimes tips for problem solving. Self-expressions of strength (eg, #type1warrior, #type1strong, #t1dstrong, and #diabadass) were used to describe individuals who were not going to let diabetes get in the way of living their best life. One person described their willingness to change their perception about their diabetes: “I'm ready to stop resenting my body, and start working with what I've got!”

Several posts included individuals engaging in healthy activities, such as exercise or eating a healthy meal. Peers encouraged others to stay on track or provided general health advice, such as drinking more water, getting enough rest, and the importance of administering a bolus before meals. Individuals who shared success stories, such as overcoming an emotional or physical barrier, were seen as inspirational. One person commented, “Happy Dexing to you, my super active diabuddy! Love to see PWD [people with diabetes] doing great things!” Recipients of these posts of encouragement wrote messages thanking individuals for their support. When individuals were experiencing inaccurate CGM, peers would inquire about recent Tylenol use or frequency of calibrations, reminding them about standard considerations for use of the CGM system.

Emojis and hashtags were used to provide encouragement and support. For example, hearts of all colors were used in a way to exhibit caring. Hand-gesture emojis, such as clapping hands, oncoming fist (ie, fist bump), OK hand (ie, index finger touching thumb to make an open circle), and victory hand (ie, peace sign) were used to exhibit concordance and approval. Hashtags were used to describe their diabetes (ie, #T1D, #type1diabetes, and #type1lookslikeme) or tools used to manage diabetes (ie, #insulinpump). Additionally, hashtags were used in fun or humorous tones (ie, #bionicwoman, #insulinjunkie, and #sexybetic).

### Continuous Glucose Monitoring-Related Frustrations

Concerns and frustrations were expressed, most often related to costs of managing diabetes or purchase of CGM devices (eg, #WhyDoTheyHaveToBeSoExpensive), failed CGM sites, hypo- or hyperglycemia that may or may not have been accompanied by physical symptoms, or feelings consistent with diabetes burnout. Insurance coverage and out-of-pocket costs were commonly asked about and shared so commenters could draw comparisons. Some individuals expressed their desire to start or restart CGM but were unable to do so because of cost. In other instances, individuals described a willingness to continue use of CGM despite cost and other frustrations they may have experienced because of the assurance CGM provided to them and their loved ones. One commenter noted, “It breaks my heart when my Dex rips out, its like money down the drain.” Failed sensors were not only financially challenging, but also emotionally challenging as one has to troubleshoot both technology and glucose readings simultaneously.

CGM accuracy, at times, was concerning. One person described, “I got the dreaded ‘???’ yesterday and Dex never recovered...I think I was overcalibrating. Sometimes I have a love/hate relationship with my Dex.” Individuals also described “compression lows” as possible causes for inaccurate CGM readings. Some described frustrations from not learning about the impact of sensor compression on glucose levels from their health care provider. Peers suggested avoiding laying on the CGM device (ie, sleeping on the alternate side of the CGM device) as one solution. Use of alternative CGM site placements was also described as a possible option.

Although mentions of diabetes distress or burnout were uncommon, when they did occur, they were accompanied by reasons for not starting, stopping, or continuing CGM; comments of mutual understanding from those who had experienced similar feelings in the past; and motivational messages from peers. A play-on-words hashtag, #duckfiabetes, was also used to express frustrations related to diabetes.

### Life Hacks to Optimize Continuous Glucose Monitoring Use

Instagram users shared diabetes *life hacks* to enhance the use of CGM. For example, individuals posted that if CGM failed in less than 7 days, they should directly contact the CGM company to request a sensor replacement. Discussions took place related to “precooking” or “soaking” the sensor in order to improve accuracy. This means placing a new sensor on the body for several hours prior to starting the official warm-up period on the device. Users reported that the extended warm-up time improves CGM accuracy, addressing that the lower accuracy may be seen within the first 24 hours of CGM use. Some individuals noted concern about the adhesive not working for the full 7-day period or experiences where the CGM device had become inadvertently unattached from the body. The use of various adhesives (ie, GrifGrips, Opsite Flexifit, and medical tape) and adhesive barrier wipes (ie, Skin Tac) were described as a way to improve CGM device adherence to the skin. One person stated, “My [adhesive] stood up well despite my sweating like a pig while running 6 miles.” The addition of adhesives were also used to extend the use of the CGM device. Concerns about CGM device adhesive causing rashes were described. One person stated, “My stomach is at the point that I need to take a break from the Dexcom for the first time in almost 2 years.” In response to concerns about rashes related to CGM device adhesive, several barrier solutions were suggested to prevent skin reactions (ie, Johnson & Johnson Tough Pads and 3M Cavilon No Sting Barrier Film). Off-label use of FLONASE on the skin prior to CGM site insertion was also described as a possible solution to preventing CGM device adhesive-related rashes.

For some, making CGM fun by accessorizing the sensor and receiver helped with the psychosocial aspect of having diabetes. Some individuals shared pictures of personalizing their CGM device with designs as a way to demedicalize the medical device. Extending the wear of the CGM device was also a described benefit. One person commented, “Oh girl! You gotta get some [personalized adhesive]! They help keep your Dexcom or pump sites on longer, AND they come in all kinds of fun shapes and colors!” Personalization occurred with the transmitter or receiver (ie, #PumpPeelz), adhesive overlay (ie, #GrifGrips), and the CGM receiver (ie, #Tallygear).

### Sharing and Learning About Off-Label Continuous Glucose Monitoring Activity

Off-label use of CGM was described as solutions to enhance CGM accuracy, decrease cost, and improve comfort. Two types of off-label activity were discussed: CGM device wear on locations other than the abdomen and extension of CGM device wear beyond 7 days. Off-label CGM activity discussions included those who had engaged in off-label activity often and those who had not. Those who had not engaged in off-label activity sometimes reported that they had only been taught the FDA-approved way of wearing the CGM device and had not thought to self-experiment but sought more information. Some parents expressed that photos viewed on Instagram would be used to explore new CGM site options with their children; one parent stated, “I'm glad you posted a photo [of a CGM site on the thigh]! I've been trying to talk my kiddo into putting it on her leg!” However, use of CGM devices on body parts other than the abdomen were oftentimes viewed as exciting and useful and, at times, more practical, less painful, and more accurate. Those who were already using CGM in off-label locations had typically experimented in more than one off-label location until they found one or more sites that were both comfortable and provided accurate readings. On person noted, “I wish my stomach was accurate. If so, it would be my favorite. Alas, my arm is my old faithful.” Sometimes, different sites were used based on the activity the individual planned to engage in. One person commented, “It all depends on activity. My arm is best when I go mountain biking or skiing but not great for arm-balancing yoga exercises.” Comfort was important as well; one person noted, “I always wear my CGM [device] on my thigh! Best spot for working out and comfort for sure!” In other instances, off-label CGM sites were used for practicality. One parent commented, “My 19-month-old daughter loves hers on the back of her arm!! Out of sight out of mind.” Few individuals new to off-label CGM site locations expressed unwillingness to show their CGM device in a location that was more publicly visible (ie, arm); many individuals only wearing the CGM device on the abdomen indicated that they planned to engage in off-label CGM wear in the future. In response to seeing a photo of a CGM device on a forearm, one person commented, “Maybe I'll try this spot next time I'm due to change [my sensor]!! I'm always open for new spots to put my Dex.” Only one post expressed unwillingness to try off-label CGM site locations, due to it not being FDA approved.

Details on how to extend the use of the CGM was described in multiple posts and comments. Those who were extending the use of CGM for more than 7 days were often proud of this accomplishment. One person noted a new record in wearing the CGM device: “Dexy finally bit the dust today after a beautiful 18 days together.” Curious about this activity, learners sought more specific details from users experienced with this activity. One commenter asked, “How do you make it last 2 weeks? My sensor always tells me I have to change after 7 days.” In response to a request for more information such as this, other commenters would describe the process of resetting the sensor on the receiver without actually removing the sensor from the body and sometimes describe how long they are able to wear a sensor. One person commented, “We leave ours in for 3-4 weeks. Just stop and restart the sensor.” Individuals stated that they extended their use of CGM beyond 7 days for several reasons. First, to save money; CGM was noted to be expensive and not having to change the CGM sensor on a weekly basis allowed them to require fewer sensors each month. One representative quote states, “I’m about to the enter my 4th week with this sensor. #WhyDoTheyHaveToBeSoExpensive.” Additionally, improvement in readings with CGM device wear over a longer period of time was also noted. One person posted, “My back and upper butt gives me the best and most accurate readings for 3-4 weeks.” Similar to off-label locations, several 7-day CGM users expressed interest in extending CGM device wear time in the future.

## Discussion

### Principal Findings

This is the first study, to our knowledge, that uses photosurveillance, a novel methodology, to examine the real-world use of CGM. We found that CGM users in this sample were successfully engaging in off-label activity related to CGM use in order to improve their experience with the technology. CGM use in location sites that were off-label yielded, in some instances, better success than FDA-approved locations in this sample of Instagram posts and comments reviewed. CGM device wear time was also extended beyond the FDA-approved 7-day period by some individuals. Instagram was used as a resource to provide and reciprocate emotional support and learn about life hacks to optimize CGM use, which included both FDA-approved and off-label activity. Our qualitative findings have several research and clinical implications.

### Photosurveillance Methodology

We successfully used photosurveillance, a new method to explore socially shared photographs, to examine real-world trends in diabetes care. This study allowed for the capture of important qualitative metrics of off-label CGM activity that otherwise may be difficult or impossible to capture in the clinical setting. As such, photosurveillance may be a beneficial methodology to explore health-related topics that may be considered taboo. In the future, other research methods may be combined with photosurveillance to examine the spread of health behavior, such as off-label CGM activity, within a network as has been done with other health conditions [10,11].

Hand-searching Instagram was a labor-intensive process. Future research should explore machine learning techniques to examine photographs and corresponding text from social media sources on a larger scale. For instance, computer vision algorithms can be leveraged to automatically process images to identify when individuals are wearing their devices in off-label locations. In particular, convolutional neural networks [24] achieve state-of-the-art accuracy for several computer vision tasks, including object recognition, object detection, and scene labeling [25-28]. Labeled Instagram posts that have been manually annotated by human coders can be used to provide training data to calibrate the model and as test data to evaluate the trained model’s accuracy.

Other types of social media platforms, including Twitter, can be utilized to examine public opinion and sentiment around CGM; these data can be processed using natural language techniques developed within the field of computer science. Machine learning and deep learning algorithms can potentially decrease the cost of research and enable research to be conducted on a larger scale. However, they may not be able to provide as detailed an analysis on features of an image or text that human coders can provide. While a human coder can evaluate potentially hundreds of characteristics of an image or scene, separate algorithms may need to be built for each characteristic to be extracted.

### Off-Label Use of Continuous Glucose Monitoring

Information about off-label use of CGM is being shared on Instagram. In this study, individuals wore CGM devices in off-label locations. A small study in a pediatric population found no accuracy difference in sensors worn on the abdomen and buttocks, which are both approved by the FDA in children, and the arm, which is off-label [29]. Further, sensor failure was equal between off-label and FDA-approved locations.

Dexcom is typically used by individuals who are dependent on insulin and therefore need to be protective of the available “real estate” on their body to optimize insulin absorption. The risk of scar tissue and the need to rotate insulin injection and pump sites may increase the desire for multiple areas to place the CGM sensor in addition to the abdomen. Recent research [30] indicates that CGM device placement in areas of lipohypertrophy, which may not necessarily be on the abdomen, has equivalent or superior glucose accuracy to that of normal tissue; however, long-term data are lacking. Currently, 68%-71% of CGM users wear their CGM device at least 75% of the time [31,32]; it is possible that use of off-label CGM locations may increase this number.

We found instances in which CGM devices were being worn successfully beyond the FDA-approved 7 days. While the sample size was relatively small (40/353, 11.3%), the qualitative data indicates that this phenomenon not only contributed to cost savings, but also improved CGM accuracy over time. With the recent FDA approval of the Dexcom G6 in the United States, which is approved for 10-day wear, the extension of use remains to be seen.

### Limitations

The interpretation of results should be considered in the context of the study limitations. It is unknown how many people with diabetes use Instagram; therefore, findings are not generalizable. In general, there were few (353/2923, 12.08%) CGM sites identified in the overall sample of photographs screened. While this does not reflect inability to capture data of CGM site placement, it does warrant the question of how valuably individuals perceive the sharing of CGM site photos, compared to other types of CGM photos, with others. Photographs only capture a snapshot in time and may not accurately reflect whether or not CGM was actually used successfully for the full length of the sensor time frame. It is possible we did not capture all reports of off-label CGM activity due to limiting the analysis to only one hashtag, #dexcom, and in only analyzing photographs of actual CGM sites. However, this study provides preliminary evidence that off-label use of CGM exists and appears to be working physiologically, while enhancing flexibility and accuracy for patients. Self-report of CGM accuracy may not reflect actual accuracy of CGM glucose levels. However, our study does confirm previous work [29] focused on glucose accuracy in off-label locations. To optimize representativeness of our sample, data was collected daily throughout the study period. Due to an inability to assess demographics of Instagram users, we did not dichotomize the data into photographs of adults and children due to risk of error. Therefore, we did not differentiate between photos of children and those of adults in our study. Children do have FDA approval to wear the Dexcom on the abdomen and buttocks, while adults only have FDA approval to wear it on the abdomen. In our study, photographs on the buttocks, a sensitive area of the body, were limited. Additionally, 10% of the sample were categorized as unknown. Future work should make an effort to examine off-label use of CGM derived from survey and/or clinical research.

### Conclusions

In this study we used photosurveillence to successfully identify real-world trends in CGM device wear, including placement and length of wear. We found individuals successfully used CGM in off-label locations with greater frequency than on the abdomen, with no indication of sensor failure or significant adverse effects. People with diabetes who are using CGM are finding ways to effectively use the tools in a manner that fits with their individual needs and goals, which is augmented by others on social media. Health care providers, industry, researchers, and even the FDA may want to utilize photosurveillance as a method to discover how people are using diabetes technologies successfully in their daily lives and perhaps adjust care protocols or product design accordingly.

## Abbreviations

CGM: continuous glucose monitoring
FDA: US Food and Drug Administration
PWD: people with diabetes
